# Cryo-EM structure of the CFA/I pilus rod

**DOI:** 10.1107/S2052252519007966

**Published:** 2019-07-09

**Authors:** Weili Zheng, Magnus Andersson, Narges Mortezaei, Esther Bullitt, Edward Egelman

**Affiliations:** a Department of Biochemistry and Molecular Genetics, University of Virginia, Charlottesville, VA, USA; b Department of Physics, Umeå University, Umeå, Sweden; c Physiology and Biophysics, Boston University School of Medicine, 700 Albany Street, Boston, MA 02118, USA

**Keywords:** fimbriae, bacterial adhesion, helical reconstruction, force spectroscopy, electron cryomicroscopy, 3D reconstruction, 3D image processing, integrative structural biology

## Abstract

Enterotoxigenic *Escherichia coli* (ETEC) are common agents of diarrhea for travelers and a major cause of mortality in children in developing countries. To attach to intestinal cells ETEC express colonization factors, among them CFA/I, which are the most prevalent factors and are the archetypical representative of class 5 pili. The helical quaternary structure of CFA/I can be unwound under tensile force and it has been shown that this mechanical property helps bacteria to withstand shear forces from fluid motion. We report in this work the CFA/I pilus structure at 4.3 Å resolution from electron cryomicroscopy (cryo-EM) data, and report details of the donor strand complementation. The CfaB pilins modeled into the cryo-EM map allow us to identify the buried surface area between subunits, and these regions are correlated to quaternary structural stability in class 5 and chaperone–usher pili. In addition, from the model built using the EM structure we also predicted that residue 13 (proline) of the N-terminal β-strand could have a major impact on the filament’s structural stability. Therefore, we used optical tweezers to measure and compare the stability of the quaternary structure of wild type CFA/I and a point-mutated CFA/I with a propensity for unwinding. We found that pili with this mutated CFA/I require a lower force to unwind, supporting our hypothesis that Pro13 is important for structural stability. The high-resolution CFA/I pilus structure presented in this work and the analysis of structural stability will be useful for the development of novel antimicrobial drugs that target adhesion pili needed for initial attachment and sustained adhesion of ETEC.

## Abstract 2

The structure of a common virulence factor expressed on the surface of diarrhea-causing bacteria, CFA/I pili, has been determined at 4.3 Å resolution. The role of Pro13 in stabilizing the pilus structure has been investigated using force-measuring optical tweezers on wild-type and point-mutated pili.

## 1. Introduction   

Enterotoxigenic *Escherichia coli* (ETEC) bacteria are common agents of infectious diarrheal diseases that cause disease in over 200 million people each year, with over 18 000 deaths of children younger than five years of age and over 50 000 total deaths (Khalil *et al.*, 2018 ▸). To infect a host, ETEC first needs to adhere, a step that is mediated by adhesion pili (also called ‘fimbriae’) (Anantha *et al.*, 2004 ▸). CFA/I pili, the archetype of class 5 pili, are the most prevalent ETEC colon­ization factor and extensive evidence indicates that either CFA/I pili or constructs of pilin subunits work as protective antigens (Freedman *et al.*, 1998 ▸; Luiz *et al.*, 2015 ▸), and the use of pilin subunits is a current strategy for vaccine development (World Health Organization, 2019 ▸).

Adhesion pili subunits are assembled into helical polymers after being chaperoned across the periplasm, transported through an usher protein that resides in the outer membrane, and pilins are then sequentially added to the growing pilus filament. This pilus assembly occurs via a ‘donor-strand exchange’ during which the N-terminal extension (NTE) of each pilin subunit is transferred from the chaperone protein to a growing chain of pilin subunits (Choudhury, 1999 ▸; Sauer, 1999 ▸; Verger *et al.*, 2007 ▸), filling an empty groove in the previous subunit (Soto & Hultgren, 1999 ▸). While the chaperone proteins for CFA/I pili have no sequence homology to the originally described chaperone–usher pathway adhesion pili, the assembly, pilus structure and function of CFA/I pili are similar to P-pili and type 1 pili, and have therefore been designated the ‘alternate chaperone pathway’ (Sakellaris & Scott, 1998 ▸). The bioassembly of CFA/I pili is initiated by the attachment of the chaperone/minor pilin (CfaA/CfaE) heterodimer to the usher protein CfaC at the outer membrane assembly site followed by cyclic incorporation of the major pilin, CfaB (Poole *et al.*, 2007 ▸; Li *et al.*, 2009 ▸). Both pilin subunits, CfaE and CfaB, require the chaperone CfaA for folding and stability and the CfaC usher protein to facilitate donor-strand exchange. This growing chain of subunits forms a helical structure 1–3 µm in length and with an outer diameter of approximately 8 nm (Mu *et al.*, 2005 ▸, 2008 ▸).

Similarly to other adhesion pili expressed by ETEC and *E. coli* that cause urinary tract infections, for example CS2, CS20, type 1 and P-pili, the quaternary structure of CFA/I can be unwound by tensile force (Andersson *et al.*, 2012 ▸; Mortezaei, Singh *et al.*, 2015 ▸; Mortezaei, Epler *et al.*, 2015 ▸; Andersson *et al.*, 2007 ▸). Tensile force sequentially unwinds subunits in the quaternary structure into a linearized open-coiled structure, thus producing a constant force response. Via physical simulations, it has been shown that when a bacterium is exposed to fluid shear forces, the capability to unwind pili assembled via donor-strand exchange reduces the load on the adhesin expressed at the tip (Zakrisson *et al.*, 2012 ▸, 2015 ▸). Unwinding, which is a mechanical property, is therefore suggested to help bacteria to attach and stay attached to host surfaces under fluid flow. Further evidence for this was shown in an *in vivo* mouse study, in which uropathogenic *E. coli* bacteria expressing point-mutated type 1 pili (also a donor-strand exchange pilus type) that required a reduced force to unwind the quaternary structure were significantly attenuated in bladder infection and intestinal colonization (Spaulding *et al.*, 2018 ▸). Thus, this study demonstrated a reduction in the intestinal reservoir of bacteria with pili that unwind under lower force, compared with bacteria expressing wild-type pili. ETEC, including those expressing CFA/I pili, establish gut infections in physiological conditions where peristaltic reflex generates pressure, shear stress and a reverse vortex-like flow of chime, as shown by experimental data (Rao *et al.*, 1996 ▸; Schulze-Delrieu, 1999 ▸) and numerical studies (Jeffrey *et al.*, 2003 ▸). Based upon these data, it is expected that CFA/I unwinding is equally important to maintaining adhesion as it is for type 1 pili. Thus, understanding the intrinsic interactions and essential properties of pili is important to better understand their role during colonization. However, what intrinsic interactions and essential properties stabilize the CFA/I pili are not yet well established.

We show here the structure of CFA/I pili at 4.3 Å resolution. A fit of the crystal subunit into the cryo-EM map illustrates conformational differences between the known crystal structures of the major pilin subunit, CfaB (Bao *et al.*, 2016 ▸) and its structure within an assembled pilus filament. We present intrinsic properties responsible for quaternary structure stability, determined by evaluating the buried surface area of subunit–subunit interactions using the CFA/I pilus model built from the cryo-EM map.

## 2. Results   

### 2.1. CFA/I pili structure   

**Figure 1:**
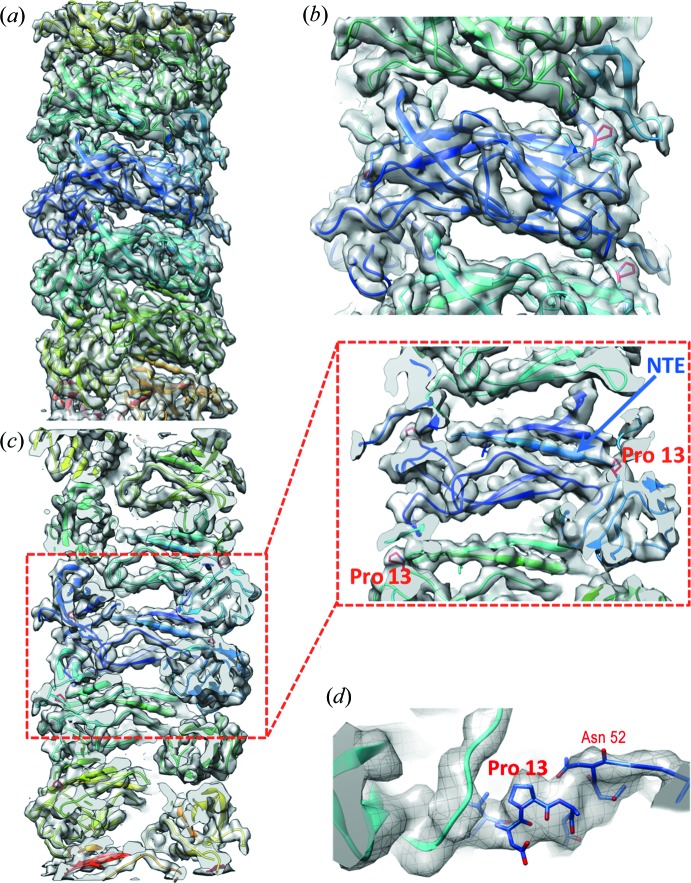
Overall reconstruction of CFA/I pili. (*a*) Side view of the CFA/I pilus reconstruction fit with the model, with subunits colored distinctively. (*b*) A representative outer region of the CFA/I pilus structure, Pro13 is highlighted in red. (*c*) The cut-away view shows core of CFA/I pilus rod, where the N-terminal extension of subunit *n* is inserted into the β-strands groove of the preceding subunit. A close-up view shows the clearly separated β-strands as well as the Pro13 at the end of N-terminal extension of each subunit. (*d*) A view of Pro13 shown in stick representation within the cryo-EM map.

**Figure 2:**
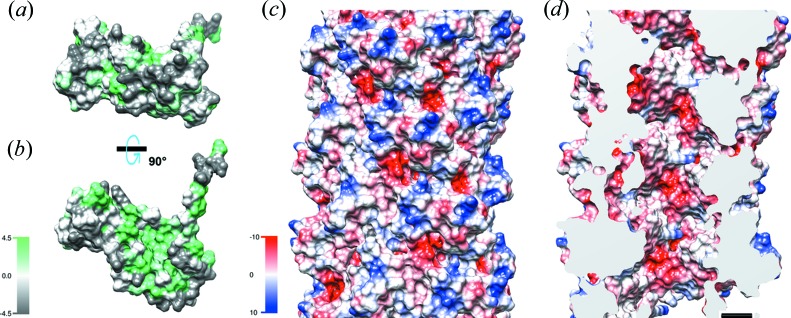
Hydrophobicity and surface charges. Hydrophobicity maps (Kyte & Doolittle, 1982 ▸; arbitrary units, from −4.5 to 4.5) of surface-exposed residues for individual subunits are shown oriented so that the outer surface (*a*, top) or the surface that accepts the N-terminal extension from the adjacent subunit (*b*, bottom) is visible. The charge distribution (in kcal mol^−1^ at 25°C) on the outer surface (*c*) and the inner surface (*d*) of the pilus shows a negatively charged inner surface that could provide a target for positively charged disruptive therapeutic molecules/peptides such as histatins (Brown *et al.*, 2018 ▸).

Our electron cryomicroscopy (cryo-EM) three-dimensional helical reconstruction of CFA/I pili [Fig. 1 ▸(*a*), Supplemetary Fig. S1] shows pilin subunits assembled into 7.8 nm diameter helical filaments with an 8.6 Å rise per CfaB subunit, and 3.18 CfaB subunits per turn of the helix. After rigid-body fitting of the subunit’s structure determined by X-ray crystallography (Li *et al.*, 2009 ▸) into the cryo-EM helical reconstruction, we used *RosettaCM* to refine the filament structure of CfaB based upon the cryo-EM map. The fit of the subunits into the cryo-EM map is shown in Figs. 1 ▸(*b*) and 1(*c*), with a ribbon backbone display. CFA/I pili are assembled using the NTE of subunit *n* fitted into the groove created by a ‘missing β-strand’ in the previously added subunit, *n* − 1. This interaction provides strong non-covalent bonds between adjacent subunits. As seen in Figs. 2 ▸(*a*) and 2(*b*), the outer face of the subunit is mostly hydro­philic [Fig. 2 ▸(*a*)], whereas there is a hydro­phobic groove visible when the subunit is rotated 90° [Fig. 2 ▸(*b*)]. This groove gets filled by the NTE of the next subunit as the pilus is assembled. Stabilization of the pilus as a helical filament appears to be primarily through contacts between subunits *n* and *n* + 3 (Fig. S2). Calculated from our CFA/I model using *CocoMaps* software (Vangone *et al.*, 2011 ▸) the buried surface area between subunits *n* and *n* + 3 is 1087 Å^2^, whereas the buried surface area between *n* and *n* + 1 is 485 Å^2^, and between *n* and *n* + 2 is 453 Å^2^. Subunit–subunit interactions that produce the buried surface area are described in Supplementary Table 1. Once assembled into the helical filament, positively and negatively charged surface residues are distributed throughout the pilus outer surface, while many negative charges are exposed on the inner surface, as seen in Figs. 2 ▸(*c*) and 2 ▸(*d*), respectively.

**Figure 3:**
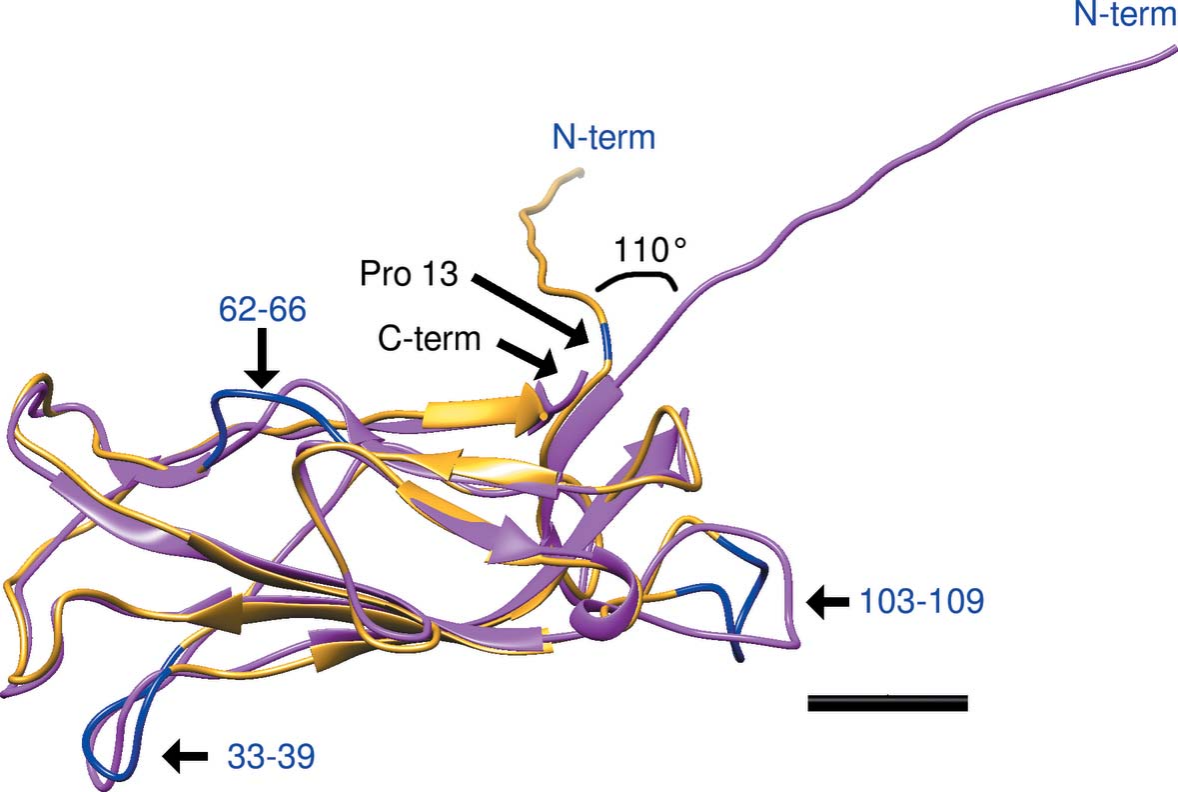
Hinge region difference. The major conformational change between subunits adopting a linear versus helical macromolecular assembly is a rotation of 110° of the N-terminal extension, residues 1–13. Additional smaller differences occur in loops at residues 33–39, 62–66 and 103–109 (marked in blue). CfaB central subunit from PDB entry 3f85, magenta; this research, yellow. Scale bar = 10 Å.

The structure of an engineered fusion protein, connecting three CfaB subunits by their NTEs after donor-strand exchange, was determined by X-ray crystallography (Li *et al.*, 2009 ▸). The most significant conformational change in the structure of CfaB in the CFA/I pilus filament, as compared to the central subunit from the CfaB trimer (PDB entry 3f85), is an approximately 110° rotation of the N-terminal β-strand (Fig. 3 ▸). In both structures, 12 NTE amino acids have an approximately linear orientation. When expressed as a trimeric oligomer and crystallized, the subsequent amino acids continue in an approximately linear orientation (Fig. 3 ▸, magenta), as is necessary for exit through the limited pore size of the usher at the bacterial outer membrane (Phan *et al.*, 2011 ▸). In contrast, an approximately 110° rotation at the end of the NTE was observed in our cryo-EM structure (Fig. 3 ▸, yellow). This rotation and the observed reorientation of the subunit beyond this point, produces a helical pilus filament. While the cryo-EM map does not have sufficient resolution to definitively assign orientations of residue side chains, the CfaB backbones are well aligned after residue 13 (Ala14, Ile15, Asp16, *etc.*), and in the pilus filament structure, there may be contacts between Pro13 of subunit *n* and Ala132 of subunit *n* − 1.

The backbone between Pro13 and Ala14 appears to bend, producing the conformational difference that is observed between the CfaB crystal structure, with its linearly arranged subunits, and the CfaB cryo-EM filament structure [Figs. 1(*d*) ▸ and 3 ▸] A visualization of how this movement might occur is shown in the supplementary movie.

Additional, smaller, conformational changes between CfaB in its fibrillar state, as compared to the CFA/I helical filament, include a reorientation of a loop from residues 103–109 (Fig. 3 ▸ and supplementary movie). In the pilus filament, this loop extends from subunit *n* − 3, occupying part of the location of the ‘staple’ that was seen previously to create interactions between subunit *n* and subunits *n* − 1, *n* − 2, *n* − 4 and *n* − 5 in the P-pilus structure (Hospenthal *et al.*, 2016 ▸). We note that in CFA/I pili there are no contacts between the subunits via this loop. As seen in Fig. 3 ▸, the orientations of two other loops within CfaB also change: residues 33–39 and residues 62–66. Point mutations in either loop 33–39 or 103–109 result in phenotypic changes in the CFA/I pilus helical filament conformation, as seen in Supplementary Fig. S3, which shows electron micrographs of negatively stained samples with Thr39 mutations to Ala or Tyr (Figs. S3A and S3B) and Ser109 mutations to Ala or Tyr (Figs. S3C and S3D).

### 2.2. Pro13 of CfaB influences quaternary structural stability under tensile force   

To investigate the impact of Pro13 on the quaternary structural stability of CFA/I pili, we used optical tweezers to measure the force required to extend individual pili. By pulling on a bead nonspecifically bound to the tip of a CFA/I pilus on a tethered bacterium, we extended the pilus from its helical to its unwound state and thereby assessed its mechanical properties. Thus, we could quantify the force needed to unwind the CFA/I helical filament.

**Figure 4:**
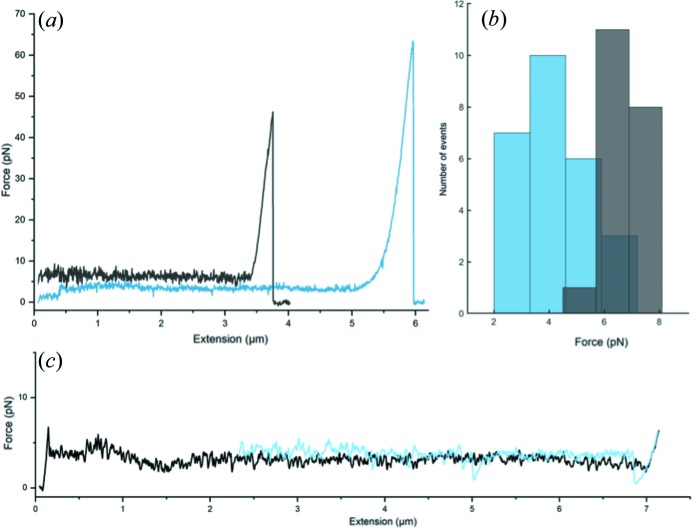
Pro13 is essential for the quaternary stability of CFA/I pili. Mutation of Pro13 to phenyl­alanine in CfaB changed the force required to unwind the quaternary structure of a pilus. (*a*) Force response of a single wild type CFA/I pilus (black) and of a single mutated CFA/I pilus (blue). (*b*) Histogram of the wild type (gray) and mutant (blue) unwinding forces. (*c*) Extension (black) and rewinding (light blue) of a single mutated CFA/I pilus.

First, we investigated the mechanical properties of CFA/I pili expressed by wild-type bacteria. We show in Fig. 4 ▸(*a*) a representative force–extension response curve (black curve) of one such pilus. The data indicate an initial linear increase in force as the helical filament stretches by approximately 10% of the filament length. The quaternary structure then unwinds, represented by the constant force plateau between 0.1 and 3.4 µm. At 3.8 µm the applied force is so high that the pilus detaches from the bead resulting in the sudden force drop.

Since the plateau force is an indicator of quaternary stabil­ity, a high unwinding force implies better stability to tensile forces. We averaged the force data of the plateaus for all measured pili, which for the wild type CFA/I was 7.0 ± 1.0 pN [mean ± standard deviation (SD), *n* = 20, two biological replicates]. Thus, these data are in agreement with previous force measurements on CFA/I (Andersson *et al.*, 2012 ▸). Next, we investigated the point mutant CFA/I pili in which Pro13 was changed to another non-polar residue, phenyl­alanine. Cells expressing these mutated structures have shown a reduced hemagglutination capability when exposed to agitation for short intervals (Li *et al.*, 2009 ▸). Also, in that study intact helical filaments were rarely observed. Thus, we hypothesized that force curves should be different than observed for wild-type CFA/I.

Indeed, our force measurements showed that this point mutation changed structural stability. Using the same force measuring approach as for the wild-type pili we found that the data for mutated pili indicate a structure that is less able to withstand tensile force [Fig. 4 ▸(*b*) (blue histogram)]. However, the mutation did not make the pilus plastic, that is, the pilus could still regain its shape by rewinding back into a helical state [Fig. 4 ▸(*c*)]. The average plateau value force was 4.5 ± 1.5 pN (*n* = 26, five biological replicates). An unpaired, two-sided *t*-test comparison between the force needed to unwind two types of pili indicates a significant difference (*p* value < 0.00001).

## 3. Discussion   

As shown previously (Li *et al.*, 2009 ▸), the CFA/I pilus filament is assembled via donor-strand exchange, where the hydro­phobic N-terminal region of an incoming subunit, *n*, fits into an available hydro­phobic groove in the previously added subunit, *n* − 1. In our new higher resolution structure, details of this interaction and the positioning of the β-strands that make up the subunit are now visualized. The CfaB subunit fits into the cryo-EM map with a 12° rotation perpendicular to the helical axis, as compared with its fit into a map produced from negatively stained pili (Mu *et al.*, 2008 ▸). In the current structure, Pro13 has a better fit in a *trans* conformation, in contrast to the expectation that a transition from *trans* to *cis* would produce the rotation required for the conformational change from a linear to a helical structure (Li *et al.*, 2009 ▸). While at our current resolution, an unambiguous fit of Pro13 into the EM map is not possible, bending of the backbone structure between Pro13 and Ala14 appears to provide the rotation necessary for assembly of the helical filament (Figs. 1 ▸ and 3 ▸). Lastly, the previously determined CFA/I pilus structure appeared to have openings from the outside surface into the central cavity, whereas at higher resolution there are surface grooves that do not extend to the pilus center (Fig. S4).

Our findings suggest that quaternary structural stability for CFA/I pili is due mainly to the stacking interface formed by every *n* to *n* + 3 contact, as demonstrated by this contact having the largest buried surface area, 1087 Å^2^ (Fig. S2). We challenged this interaction by applying tensile force using optical tweezers and measured a 7.0 ± 1.0 pN force needed to unwind CFA/I pili. This finding correlates well with what has been shown for P pili and type 1 pili as both of these pilus types have more buried area, 1453 and 1616 Å^2^ (Hospenthal *et al.*, 2016 ▸, 2017 ▸; Spaulding *et al.*, 2018 ▸), and require stronger unwinding forces (Andersson *et al.*, 2007 ▸). However, there appear to be additional subunit–subunit interactions in CFA/I pili that have a large influence on its quaternary stability. For example, mutation of Pro13 has been shown to cause significant phenotypic changes in the CFA/I pilus. Initial analysis of EM images showed that a large majority of pili observed by electron microscopy were 2–3 nm diameter fibrillar structures, rather than 8 nm diameter filamentous (helical) structures (Li *et al.*, 2009 ▸). Later experiments showed that unwinding had been increased by ‘typical’ pipetting, and more gentle treatment resulted in the observation of intact helical filament segments (Fig. S5). From our new structural and biophysical data, we expect that the increase in unwound pili in the Pro13 CfaB mutant was due to missing contact between Pro13 and the preceding subunit, *n* − 1. Without this contact, and with the additional loss of backbone rigidity that had been provided by proline, it appears to be more difficult for the pilus to coil and to maintain a helical filament. Conversely, the linear fibrillar structure would probably be uncompromized, as Pro13 does not participate in the *n* to *n* − 1 donor-strand exchange interaction. The lowered stability of CFA/I pili after mutation of Pro13 to phenyl­alanine was validated by our measurement of a lower unwinding force for the mutant CFA/I pilus (Fig. 4 ▸) and EM images showing many regions of unwound pili (Fig. S5).

The surface charge and hydro­phobicity maps of CFA/I pili show characteristic positive, negative and some hydro­phobic residues on the outer surface of the pilus. Of particular interest is the highly negatively charged inner surface of the pilus. Further studies will be used to test a mechanism for disruption of pili by positively charged antimicrobial peptides, such as histatin 5, via interaction with this negatively charged surface (Brown *et al.*, 2018 ▸). This detailed view of the structure of CFA/I pili provides an opportunity for developing new strategies to disrupt bacterial adhesion, and limit or prevent subsequent disease.

## 5. Methods   

### 5.1. Sample preparation   

ETEC bacteria E7473 were grown overnight on 20 CFA agar plates (1.0% Casamino acids, 0.15% yeast extract, 0.005% MgSO_4_, and 0.0005% MnCl_2_ 2.0% agar) with 50 µg ml^−1^ kanamycin at 37°C, overnight. Bacteria were resuspended in 25 ml of 0.5 m*M* Tris, 75 m*M* NaCl, pH 7.4 and incubated at 65°C for 30 min to extract pili. Bacteria without pili were pelleted at 10 000*g*, and the supernatant was centrifuged at 14 500*g* for 30 min to remove cell debris. Pili were precipitated in 300 m*M* NaCl and 0.1 *M* MgCl_2_, allowed to sit for 3 h, and centrifuged at 25 000*g* for 40 min. All centrifugation steps were at 4°C using an SS-34 rotor in a Sorvall RC5B centrifuge. Pili were resuspended overnight in 0.5–1 ml of 0.5 m*M* Tris, pH 7.4; precipitation and resuspension were repeated 1 or 2 times, and pili were dialyzed against 0.5 m*M* Tris, pH 7.4.

### 5.2. Cryo-EM data collection and image processing   

3 µl of sample were applied to plasma cleaned lacey carbon grids (Ted Pella, Inc., 300 mesh), followed by plunge-freezing using a Vitrobot Mark IV (FEI, Inc). The vitrified grids were imaged with a Falcon III direct electron detector (pixel size 1.09 Å per pixel) in a Titan Krios at 300 keV. A total of 2880 movies, each of which was composed of 33 frames with a total dose of ∼45 e Å^−2^, were collected using integration mode. The defocus range was set as −0.5 to −3 µm. Images were motion corrected using *MotionCor*2 (Zheng *et al.*, 2017 ▸), and the program *CTFFIND*3 (Mindell & Grigorieff, 2003 ▸) was used for estimating the defocus and astigmatism. Images with good CTF estimation as well as defocus <3 µm were selected to use in the following helical reconstruction. The helical reconstruction was performed using the *SPIDER* software package (Frank *et al.*, 1996 ▸) with the aligned first 15 frames (∼20 e Å^−2^) of the motion-corrected image stacks. CTF was corrected by multiplying the images with the theoretical CTF, which corrects the phases and improves the signal-to-noise ratio. The *e2helixboxer* routine within *EMAN*2 (Tang *et al.*, 2007 ▸) was used for boxing the long filaments from the images. A total of 117 011 384-pixel-long overlapping segments, with a shift of 12 px between adjacent segments (∼97% overlap), were extracted for further use in the *Iterative Helical Real Space Reconstruction* (*IHRSR*) (Egelman, 2000 ▸). With a featureless cylinder as an initial reference, 97 295 segments were used in *IHRSR* cycles until the helical parameters (axial rise of 8.6 Å and rotation of 113.3° per subunit) converged. Two largely non-overlapping data sets were generated by adding 100 sequential segments at a time in each data set, which were used to reconstruct two independent half maps. The resolution of the final reconstruction was determined by map:map and model:map FSC (Fourier shell correlation), which was 4.3 Å.

### 5.3. Model building and refinement   

We used the central subunit from the structure of the major pilin CfaB previously determined by X-ray crystallography (PDB entry 3f85) as an initial model and fit it into the cryo-EM map by rigid-body fitting. Model modification was performed by manually editing the model in *UCSF Chimera* (Pettersen *et al.*, 2004 ▸) and *Coot* (Emsley & Cowtan, 2004 ▸). We then used the modified model as the starting model for further iterative refinement with *RosettaCM* (Wang *et al.*, 2015 ▸) and *Phenix* (Adams *et al.*, 2010 ▸). The refined protomer model of CfaB was then re-built by *RosettaCM* with helical symmetry and real-space refined in *Phenix* to improve the stereochemistry as well as maximize model-map correlation. The final CfaB model was validated with *MolProbity* (Chen *et al.*, 2010 ▸) and the coordinates were deposited in the Protein Data Bank with the accession code 6nrv. The corresponding cryo-EM map was deposited in the EMDB with accession code EMD-0497. The cryo-EM images were deposited in EMPIAR with accession code EMPIAR-10267. The refinement statistics are given in Supplementary Table S2.

### 5.4. Optical tweezers measurements   

We carried out force spectroscopy measurements using a high-resolution optical tweezers system constructed around an inverted microscope (Olympus IX71, Olympus) with a high numerical aperture oil-immersion objective (UplanFI 100× NA = 1.35; Olympus) (Andersson *et al.*, 2006 ▸). Cells and microbeads are trapped by a single frequency CW diode pumped laser (Cobolt Rumba) that provides a Gaussian TEM_00_ mode with a wavelength of 1064 nm. As force probe, we used surfactant-free 2.5 µm white amidine polystyrene latex beads (product No. 3–2600, Invitrogen) the position of which was monitored by projecting the beam of a low-power fiber-coupled HeNe laser (1137, Uniphase, Manteca) operating at 632.8 nm onto a position-sensitive detector (L20-SU9, Sitek Electro Optics, Partille). The detector signal is amplified with preamplifiers (SR640, Stanford Research Systems) and transferred via an analog–digital (A/D) converter card (PCI 6259M, National Instrument) to the computer where quantitative analysis is performed with a customized in-house *LabView* program. The stability of the setup was analyzed prior to measurements with the Alan-variance method to minimize the noise level (Andersson *et al.*, 2011 ▸). We measured the temperature using a thermocouple coupled to the sample chamber to 23.0 ± 0.1°C. In addition, we assumed that the suspension viscosity only varied with temperature, and thus, the viscosity was set to 0.932 ± 0.002 mPa s.

We suspended bacteria in 1× PBS to a low density suitable for single cell analysis. Microspheres were similarly suspended in 1× PBS. We prepared a sample chamber by attaching two pieces of double-sided adhesive tape (product no. 34-8509-3289-7, 3M) spaced with 5 mm, on a 24.0 × 60.0 mm cover slip (No. 1, Knittel Glass). A 20.0 × 20.0 mm cover slip (No. 1, Knittel Glass) was thereafter gently positioned on top of the adhesive tape, thus forming a 5.0 × 20.0 × 0.1 mm flow channel (Mortezaei *et al.*, 2013 ▸). We infused the bacterial suspension or the silica microsphere by adding a few microlitres of suspension at one of the openings allowing capillary forces to fill the chamber. To avoid drying of the sample, we sealed the ends of the chamber with vacuum grease (DOW Corning). The sample was thereafter mounted in a sample holder that was fixed to a piezo-stage (Physik Instrument, P-561.3CD stage) in the optical tweezers instrumentation.

## Supplementary Material

Supplementary Tables S1, S2 and Supplementary Figures S1, S2, S3, S4, S5. DOI: 10.1107/S2052252519007966/pw5005sup1.pdf

The backbone between Pro13 and Ala14 appears to bend, producing the conformational difference that is observed between the CfaB crystal structure, with its linearly arranged subunits, and the CfaB cryo-EM filament structure. DOI: 10.1107/S2052252519007966/pw5005sup2.mp4

PDB reference: CFA/I pilus rod, 6nrv

EMDB reference: CFA/I pilus rod, EMD-0497
