# Supplementary material for: Cryo-EM structure of the CFA/I pilus rod
Source: IUCrJ. 2019 Jul 9;6(Pt 5):815–21. doi: 10.1107/S2052252519007966 (PMC6760452; doi:10.1107/S2052252519007966)
Supplement: Supplementary file 1 [file m-06-00815-sup1.pdf]

IUCrJ

Volume 6 (2019)

Supporting information for article:

Cryo-EM structure of the CFA/I pilus rod

Weili Zheng, Magnus Andersson, Narges Mortezaei, Esther Bullitt and Edward Egelman

Table S1 .Subunit-subunit interactions in the assembled CFA/I pilus filament

Interacting subunits	*# of interacting residues from subunit n*	*# of interacting residues from contact subunit*	*# of hydrophilic-hydrophobic interactions*	*# of hydrophilic-hydrophilic interactions*	*# of hydrophobic-hydrophobic interactions*
n to n+1	24	18	53	25	23
n to n+2	18	11	31	16	19
n to n+3	49	33	99	77	30

Table S2 Cryo-EM data collection, reconstruction and model refinement statistics

Cryo-EM Data Collection	
Microscope	Titan Krios
Voltage (kV)	300
Detector	Falcon III
Pixel size (Å)	1.09
Defocus range (µm)	-0.5 to -3
Helical Reconstruction	
Number of movies	2,880
Number of segments	117,011
Map Resolution (Å)	4.3
Map sharpening B-factor (Å2)	-220
Model Refinement and Validation	
MolProbity score	1.89
Clashscore	3.52
Poor rotomers (%)	0
Ramachandran plot	
Favored (%)	88.89
Allowed (%)	11.12
Outliers (%)	0
R.m.s. deviations	
Bond lengths (Å)	0.006
Bond angles (°)	1.483

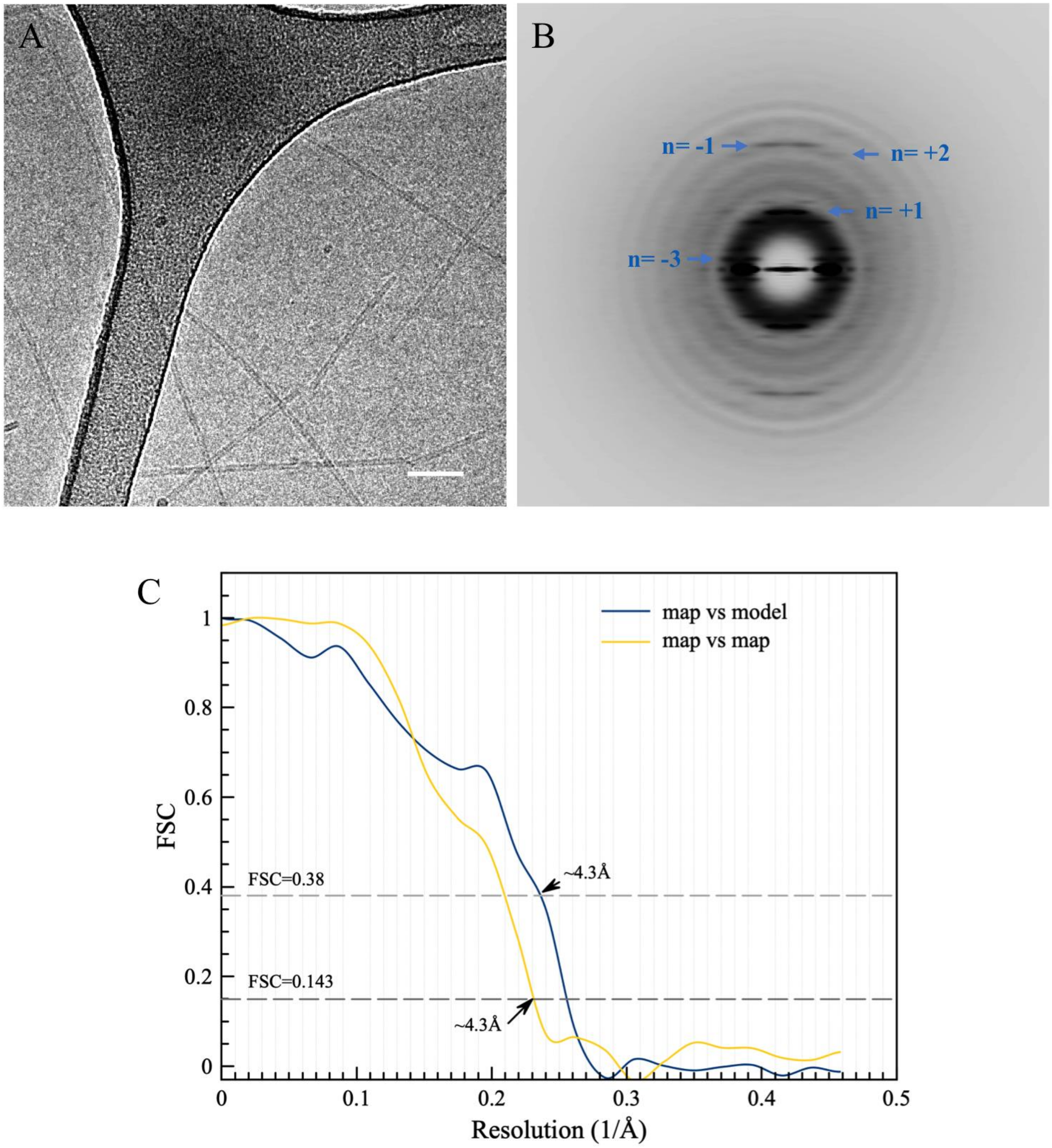

Figure S1 ***Cryo-EM reconstruction of CFA/I pili.*** (A) Representative electron micrograph of CFA/I pili in vitreous ice. Scale bar 50 nm. (B) The averaged power spectrum, generated from ~90,000 segments, that was used to determine the initial helical symmetry. The Bessel orders are labeled on four of the layer lines. (C) FSC plots for model-map (green) and map-map (yellow), showing a consistent resolution of 4.3 Å. Model-map FSC was evaluated at FSC=0.38 (which is √0.143), and map-map FSC was evaluated at FSC=0.143.

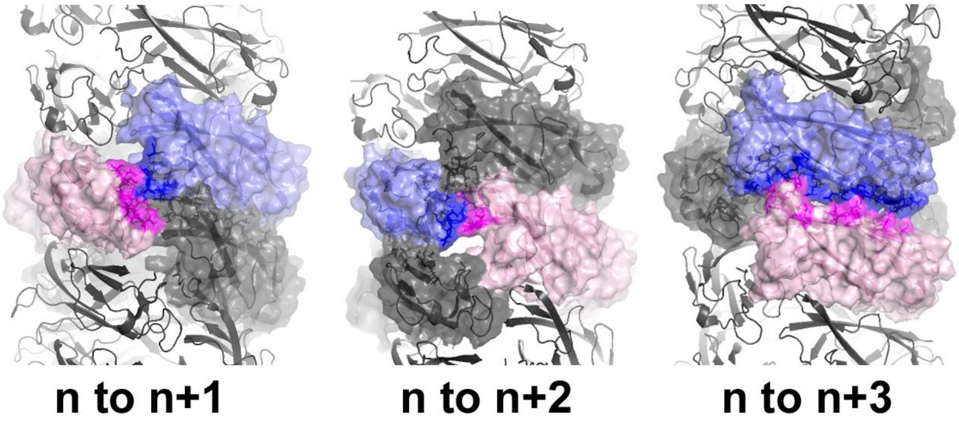

Figure S2 *Buried surface area*. The majority of the buried surface area between subunits in the assembled CFA/I pilus structure is between subunits n and n+3. Subunit n, blue; contact subunit, pink; buried surface area, magenta.

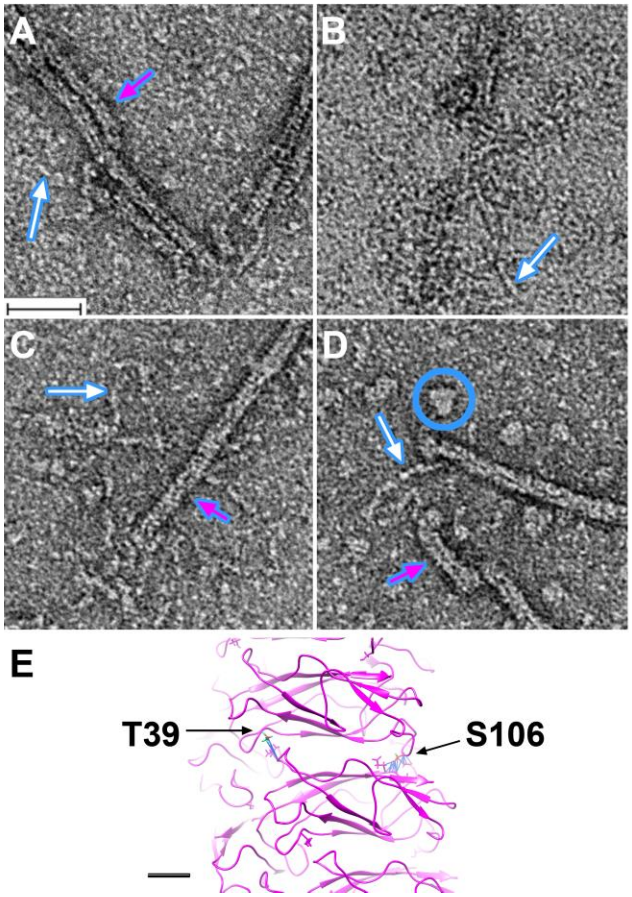

Figure S3 ***Mutations in loop regions result in phenotypic changes in CFA/I pili*.** Point mutations in loop regions that vary between the crystal structure of CfaB (pdb 3F85) and our cryoEM map reduce the stability of the helical filament. (A) Thr 39 to Ala, (B) Thr 39 to Tyr, (C) Ser 39 to Ala, (D) Ser 39 to Tyr. Magenta arrows: helical CFA/I filament; white arrows: unwound fibrillar region; blue circle: possible cross-section of a broken filament segment. Scale bar for A-D is 30 nm; scale bar for E is 1 nm.

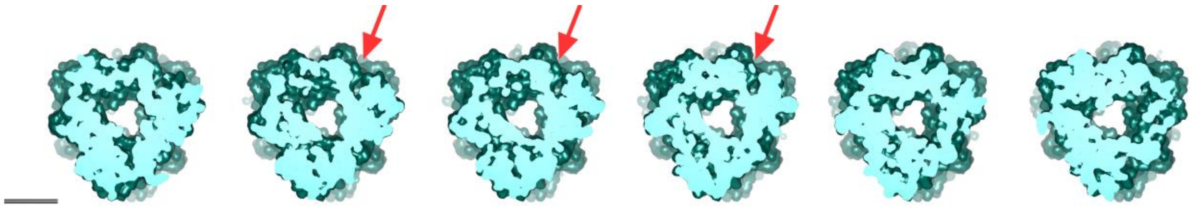

Figure S4 ***Cross-sectional views of CFA/I pili*.** Sections every 2 Å showing a groove in the pilus structure without an opening to the central cavity. Scale bar 25 Å.

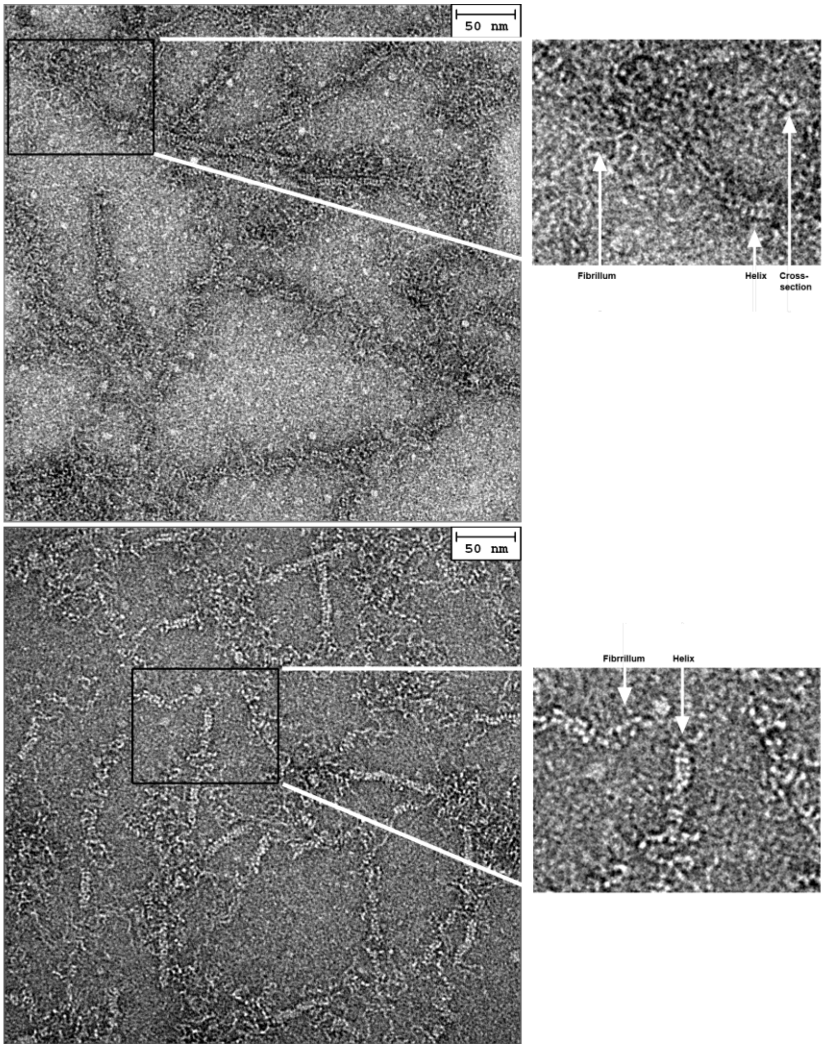

Figure S5 .***Mutation of Pro 13 results in easily unwound CFA/I pili.*** With gentle handling, pili with Pro 13 mutated to Phe can maintain regions of helical filaments. Sample was negatively stained; scale bars 50 nm, and enlarged regions are magnified 2x
